# Insights into the characteristics of primary radioresistant cervical cancer using single-cell transcriptomics

**DOI:** 10.1007/s13577-023-00882-x

**Published:** 2023-03-03

**Authors:** Biyuan Xing, Congli Pu, Yunshang Chen, Yuhan Sheng, Baofang Zhang, Jie Cui, Gang Wu, Yingchao Zhao

**Affiliations:** 1 Cancer Center, Union Hospital, Tongji Medical College, Huazhong University of Science and Technology, Wuhan, 430022 China; 2 Institute of Radiation Oncology, Union Hospital, Tongji Medical College, Huazhong University of Science and Technology, Wuhan, 430022 China; 3 CAS Key Laboratory of Molecular Virology & Immunology, Center for Biosafety Mega-Science, Institut Pasteur of Shanghai, Chinese Academy of Sciences, Shanghai, China

**Keywords:** Conditional reprogramming, Primary cell lines, Single-cell transcriptomics, Cervical cancer, Radioresistance

## Abstract

Radioresistance is a major cause of radiotherapy failure among patients with cervical cancer (CC), the fourth most common cause of cancer mortality in women worldwide. Traditional CC cell lines lose intra-tumoral heterogeneity, posing a challenge for radioresistance research. Meanwhile, conditional reprogramming (CR) maintains intra-tumoral heterogeneity and complexity, as well as the genomic and clinical characteristics of original cells and tissues. Three radioresistant and two radiosensitive primary CC cell lines were developed under CR conditions from patient specimens, and their characteristics were verified via immunofluorescence, growth kinetics, clone forming assay, xenografting, and immunohistochemistry. The CR cell lines had homogenous characteristics with original tumor tissues and maintained radiosensitivity in vitro and in vivo, while also maintaining intra-tumoral heterogeneity according to single-cell RNA sequencing analysis. Upon further investigation, 20.83% of cells in radioresistant CR cell lines aggregated in the G2/M cell cycle phase, which is sensitive to radiation, compared to 38.1% of cells in radiosensitive CR cell lines. This study established three radioresistant and two radiosensitive CC cell lines through CR, which will benefit further research investigating radiosensitivity in CC. Our present study may provide an ideal model for research on development of radioresistance and potential therapeutic targets in CC.

**Supplementary Information:**

The online version contains supplementary material available at 10.1007/s13577-023-00882-x.

## Introduction

Cervical cancer (CC) is the fourth most common malignancy among women, accounting for approximately 7% of cancer cases [1, 2]. Although relatively controlled for several decades in many high-income countries, CC remains the major cause of cancer-related death among women in low- and lower middle-income countries [3], seriously endangering women's health. Currently, treatment options for CC include surgery, radiotherapy, chemotherapy, and immunotherapy [4]. Radiotherapy is the major radical treatment option for patients with CC in stages IB3 and IIA2‒IVA, and an optional radical therapy for patients with CC stages IB1‒2 and IIA [4]. Although CC is relatively sensitive to radiotherapy, recurrence occurs within 2 years among 20–40% of patients who receive radiotherapy [5–7], with local pelvic recurrence and distant metastasis being the main recurrence patterns [6]. Patients with recurrent CC often have poor prognosis, and the 5-year overall survival is approximately 10–20% [8–10]. Additionally, patients with advanced CC are more likely to develop radioresistance [11]. Increasing doses of radiation elevates the risk of developing irradiation cystitis or enteritis, but low radiation dose cannot achieve its therapeutic effect [12]. Therefore, developing a better understanding of radiosensitivity in CC is a research hotspot for oncologists.

Most CC researches are based on traditional, established cell lines, such as SiHa, C33A, and ME180 etc. which are homologous in both morphology and phenotype and proliferate easily in vitro. However, loss of intra-tumoral heterogeneity is the main obstacle in developing drugs, including radiosensitizers, and research investigating radioresistance [13]. A comparison with transcriptomic sequencing data in The Cancer Genome Atlas (TCGA), patient-derived xenografts (PDXs), and conventional cell lines revealed that PDXs, as a substitution for patient-derived tumors (PDTs), had consistent gene expression with those of TCGA, unlike traditional cell lines [14]. In addition, conventional cell lines had poor specificity for different cancer types and their gene expression patterns differed from those of tumors, which was caused by genetic drift and clonal selection during culturing in vitro [14]. Thus, traditional CC cell lines established without receiving radiotherapy are not appropriate models for radioresistance research.

Conditional reprogramming (CR) methods have been used to culture tumors from patients with CC with the support of feeder cells and conditional medium, enabling infinite proliferation in vitro [15]. Whole-exome sequencing (WES) and copy number variation (CNV) testing of CR cell lines and tumors have demonstrated high conservative phenomena of DNA and maintenance of tumor genomic heterogeneity [16–18]. Thus, CR is used in drug screening and cancer model construction for it maintaining the characteristics of cells in vitro [17, 19]. Radiosensitive and radioresistant primary CR cell lines are needed for use as CC cell models in the study of radioresistance mechanisms, development of individualized therapies, and prognostic prediction [20, 21].

The development of high-throughput approaches, such as single-cell RNA sequencing (scRNA-seq), has enabled analysis of the molecular characteristics of individual cells in tumors. Nevertheless, the transcriptional spectrum of radioresistant and radiosensitive CC cells has not yet been investigated. The goal of current study was to establish radioresistant and radiosensitive CR cell lines from CC specimens and verified their characteristics in vitro and in vivo. For the first time, this study proclaims the molecular characteristics and biological changes of radioresistant and radiosensitive CC cells via scRNA-seq, providing a greater understanding of radiosensitivity that will benefit predictions of prognosis and radiotherapy response.

## Materials and methods

### Patient specimens

Three radioresistant CC specimens were collected from local recurrent lesions of patients who received radiotherapy within 12 months and without re-irradiation or other treatments. Two radiosensitive CC specimens were collected from patients at diagnosis who achieved clinical remission after radiotherapy and remained disease-free survival for at least 12 months.

### Establish primary cell lines by conditional reprogramming

CC tumor tissues were excised and cell suspensions were prepared following the protocol described by Liu et al. [15]. Briefly, tissue was minced and digested by 0.1% collagenase type IV (Sigma-Aldrich, USA) solution for 1 h in 37 °C incubator. Then, the cell suspension was filtered through 100 µm cell strainer into a centrifuge tube and centrifuged at 1000 rpm for 5 min at 4 °C. The cells were plated with irradiated Swiss-3T3-J2 feeder cells in a T25 flask containing complete F medium at 37 °C in a 5% CO_2_ incubator. All experiments were performed using mycoplasma-free cells. After reaching 80% confluence, the primary cells were passaged with 0.02% EDTA (Procell, China) and 0.05% trypsin/EDTA (Procell, China) consequently.

Complete F medium was prepared using DMEM (Gibco, USA), 10% fetal bovine serum (Gibco, USA), 1% 100 × Pen-Strep (Biosharp, China), 1% 100 × L-glutamine (Biosharp, China), 5 µg/mL insulin (Biosharp, China), 250 ng/mL amphotericin B (Biosharp, China), 10 µg/mL gentamicin (Biosharp, China), 0.1 nM cholera toxin (MedChem Express, China), 0.125 ng/mL EGF (Peprotech, USA), 25 ng/mL hydrocortisone (Biosharp, China) and 10 µM ROCK inhibitor Y-27632 (AbMole Bioscience, USA).

### Morphology and growth curve

Cellular morphology was examined and photographed using a phase-contrast microscope (Olympus, Japan). The population doubling times were calculated for cells in early passages. Briefly, 1 × 10^4^ cells were seeded onto a 6-well plate in triplicate, and cell numbers were counted at 1, 3, 5, and 7 days. The growth rate and population doubling time were calculated from the growth curves.

### Clonogenic assay

Equal numbers of cells were plated in triplicate onto 6-well plates for 24 h, then received radiotherapy of 0, 2, 4, 6, 8 Gy. The plates were incubated at 37 °C for 2–3 weeks. Then washed with phosphate-buffered saline (PBS), fixed with 4% paraformaldehyde, and stained with 0.05% crystal violet. Colonies with > 50 cells were counted and photographed.

### Short tandem repeat (STR) profiling

Genomic DNA from primary cell lines and corresponding peripheral blood samples of patients was isolated using the Tissue DNA Kit (Omega, China). Matching comparison of DNA fingerprinting including a total of 21 STR loci (D5S818, D13S317, D7S820, D16S539, VWA, TH01, AMEL, TPOX, CSF1PO, D12S391, FGA, D2S1338, D21S11, D18S51, D8S1179, D3S1358, D6S1043, PENTAE, D19S433, PENTAD, and D1S1656) was conducted between peripheral blood samples and CR cell lines, as well as between the CR cell lines and data for traditional cell lines in the American Type Culture Collection (ATCC), Deutsche Sammlung von Mikroorganismen und Zellkulturen (DSMZ), Japanese Collection of Research Bioresources (JCRB) and RIKEN databases.

### Xenograft models in nude mice and immunohistochemistry (IHC) straining

The animal research was performed under the NIH Guidelines for the Care and Use of Laboratory Animals and supervised by the Animal Care Committee of Tongji Medical College. In vivo primary cell lines were injected into 4–6 weeks old BALB/c nude mice (Bioscience, China). Briefly, subcutaneous injections of 5 × 10^6^ cell suspensions which were prepared in 100 μL DMEM were conducted on left flank of the hindleg of anesthetized female mice. Injected mice were observed and the tumors were examined every three days. IHC straining was performed following the general procedure using the IHC kit (Biossci, China).

### Immunofluorescence

A total of 5 × 10^4^ cells were seeded on a chamber slide (Nest Scientific, China) in a 24-well plate for 24 h, followed by washing 3 times with PBS before being fixed with 4% paraformaldehyde. After permeabilization with 0.3% Triton X-100 for 15 min, the cells were incubated with 5% bovine serum albumin (BSA) for 1 h at room temperature. Then cells were covered with primary antibodies against CK5 (1:200, Abcam, UK), Ki67 (1:200, Proteintech, USA), p63 (1:200, Proteintech, USA), and p16 (1:100, ABclonal, China), respectively, overnight at 4 °C. After 3 times washes, the secondary antibody (Servicebio, China) was added on the slides for 1 h. Nuclei were stained with DAPI (Biosharp, China). Chamber slides were analyzed and imaged by fluorescence microscope (Olympus, Japan).

### ScRNA-seq analysis

Raw disembarkation data quality statistics, cell ranger, cell refiltration, dimensionality reduction and clustering, differential genes and functional enrichment analysis, pseudo-time trajectory analysis, gene set variation analysis, CNV evaluation and transcriptional factor prediction were described in the Supplementary Methods (Online Resource 1. Methods and Materials).

### Statistical analysis

Statistical significance was determined by the Student’s t-test or two-way ANOVA using GraphPad Prism 7.0 software (GraphPad Software, USA), and presented as *p < 0.05, **p < 0.01, ***p < 0.001, ****p < 0.0001. Results were represented as the mean ± standard deviation (SD) or mean ± standard error of the mean (SEM). Data shown were representatives of at least three independent experiments, which showed similar results.

## Results

**Fig. 1:**
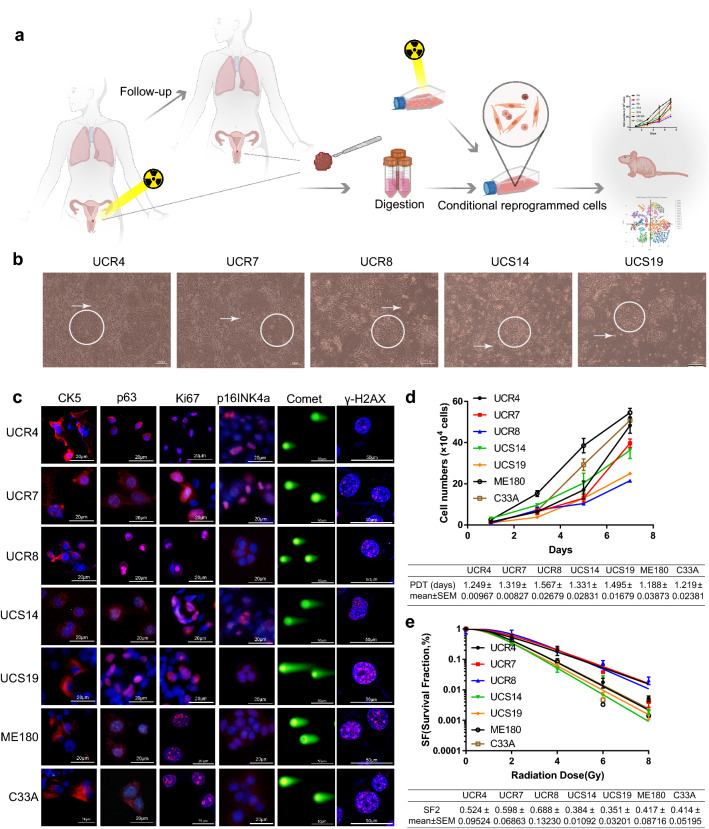
Morphology and characteristics of CR cell line. **a** Brief workflow of the establishment of CR cell lines. **b** Morphology of CR cell lines derived from patient specimens. 100 × magnification. **c** Expression of biomarkers, Comet Assay, and γ-H2AX Foci of CR and traditional cell lines. **d** Growth curve and population doubling time of different CR cell lines compared to those of traditional cell lines ME180 and C33A. Data represented mean ± SD of each group (*n* = 3). **e** Radiosensitivity (SF2 values) of different CR cell lines compared to that of traditional cell lines ME180 and C33A. Data represented mean ± SD of each group (*n* = 3). PDT, population doubling time

The primary radioresistant CR cell lines (Union Hospital Cervical Cancer Radioresistant (UCR) 4, UCR7, and UCR8) and radiosensitive CR cell lines (Union Hospital Cervical Cancer Radiosensitive (UCS) 14, and UCS19) formed cancer nests when cultured with irradiated feeder cells (Fig. 1a). All CR cell lines exhibited a monolayer polygonal shape, indicating that the tumors were squamous cell carcinomas (Fig. 1b). As shown in Table 1, the CR cell lines expressed CK5 and p63 (markers of squamous cell carcinoma), Ki67 (a proliferation index), and p16 (related to HPV infection) (Fig. 1c).

**Table 1:** Brief clinical information of patients with cervical cancer from whom CR cell lines were established

	CR cell lines				
Characteristics	UCR4	UCR7	UCR8	UCS14	UCS19
Age at diagnosis	56	56	69	67	46
Histological type	SCC	SCC	SCC	SCC	SCC
HPV type	HPV58	HPV16	HPV16	HPV16	HPV16
Tumor size at diagnosis (mm)	47 × 20	11 × 8 × 7	26 × 39 × 36	25 × 30 × 24	26 × 28
Clinical stage at diagnosis (FIGO 2018)	IIIB	IVB	IIIA	IIIC1	IIIC1
Lymph node metastasis	None	Inguinal and retroperitoneal lymph node	None	Pelvic lymph node	Pelvic lymph node
Radiotherapy					
EBRT	PCTV 50.4 Gy/28F	PCTV 50.4 Gy/28F, PGTVnd 60 Gy/28F	PCTV 50.4 Gy/28F, PGTVnd 56 Gy/25F	PCTV 50.4 Gy/28F, PGTVnd 60 Gy/28F	PCTV 50.4 Gy/28F, PGTVnd 62 Gy/28F
Brachytherapy	HRCTV D90 29.2 Gy/4F	HRCTV D90 33.4 Gy/5F	HRCTV D90 28.4 Gy/4F	HRCTV D90 27.6 Gy/4F	HRCTV D90 27.7 Gy/4F
Survival					
PFS of radiotherapy (months)	9	1	11	19	14
OS of radiotherapy (months)	–	3	32	–	–
Follow-up period (months)	–	3	32	–	–

*SCC* squamous cell carcinoma; *EBR*T external beam radiation therapy; *PFS* progression-free survival; *OS* overall survival

The CR cell lines grew in an adherent manner with different population doubling times (Fig. 1d). The CR cell lines also maintained the characteristics of tumor radiosensitivity in vitro, for more double-strand breaks and γ-H2AX foci shown in radiosensitive CR cell lines compared with radioresistant ones after irradiation (Fig. 1c). The results of the clonogenic assay (SF2 values) were consistent with the conclusion (Fig. 1e).

The 21 STR loci were homogenous between the CR cell lines under passage 10 and parental peripheral blood samples, indicating that the CR cell lines maintained the genetic information of patients when cultured in vitro (Online Resource 1. Table 1). However, the STR loci in the CR cell lines differed from those of traditional cell lines reported in the ATCC, DSMZ, JCRB, and RIKEN databases.

**Fig. 2:**
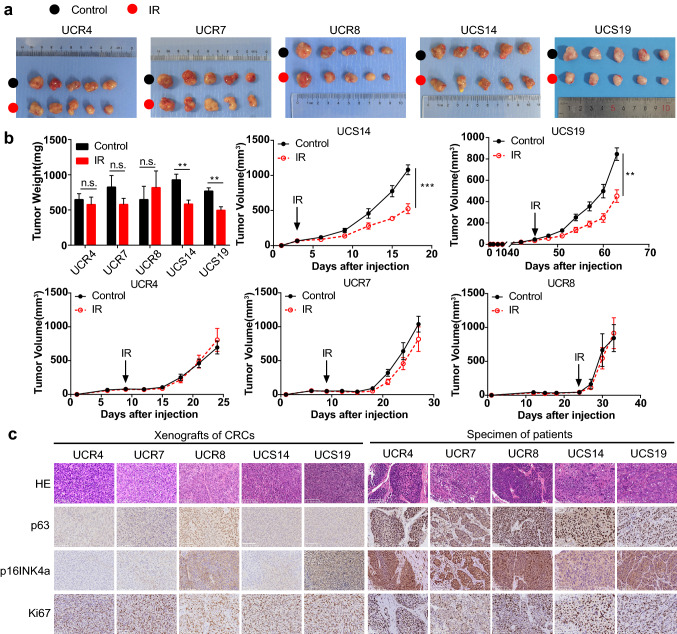
CR cell lines maintain tumorigenicity and radiosensitivity in vivo. **a** Representative images of xenografts of UCR4, UCR7, UCR8, UCS14, and UCS19. **b** Weights and volumes of xenografts after irradiation. Data represented mean ± SEM of each group (*n* = 5). **c** H&E staining and expression of biomarkers for xenografts and specimens from corresponding patients. 200 × magnification

Next, xenografts of CR cell lines were established in mice and their tumorigenicity was verified. The xenografts kept the radiosensitivity of PDTs after irradiation (Fig. 2a, b), exhibiting positive expression of p63, Ki67, and p16, which aligned with the results for the PDTs (Fig. 2c). Thus, the xenografts of CR cell lines maintained the characteristics of tumors and were an alternative model for oncology research.

**Fig. 3:**
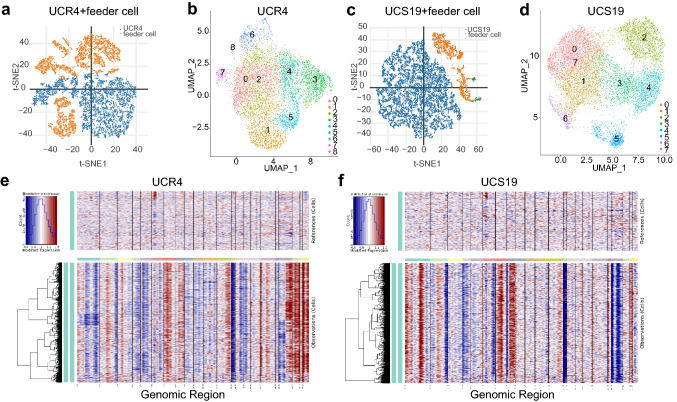
CR maintains intra-tumoral heterogeneity in cell lines derived from CC tumors. **a, c** The t-SNE plots presenting the mixture of human CR cells and marine feeder cells for UCR4 and UCS19, respectively. Blue, UCR4 or UCS19; orange and green, feeder cells. **b, d** The UMAP plots demonstrating main cell cluster types in UCR4 and UCS19. **e, f** Hierarchical heatmaps showing large-scale CNVs of UCR4 and UCS19 compared to normal tissue

To investigate the crucial oncogenes or pathways associated with radiosensitivity in CC, CR cell lines UCR4 and UCS19 were selected to conduct scRNA-seq using 10 × Genomics Chromium platform. High-dimensional data were visualized into low dimensional space by t-distributed stochastic neighbor embedding (t-SNE). Cells with similar characteristics were classified into a cluster according to the algorithm of t-SNE, and the identified clusters were displayed. A mixture of human CR cell lines and murine feeder cells were captured (Fig. 3a, c). A total of 11,808 cells for UCR4 and 12,208 cells for UCS19 were mapped to human genome CRCh38 (excluding those mapped to murine genome mm10) and investigated during the following analysis. Graph-based clustering identified 9 and 8 cell clusters in UCR4 and UCS19, respectively (Fig. 3b, d). The different gene expression patterns of cell clusters in UCR4 and UCS19 indicated that CR cell culturing maintained intra-tumoral heterogeneity of the primary cell lines to some extent (Online Resource 1. Figure 1b, f), which truly presented the characteristics of tumor and was a more appropriate approach to study radiosensitivity compared with traditional cell lines. In addition, CNV analysis of UCR4 and UCS19 compared to normal tissue further verified the heterogeneity of the CR cell lines (Fig. 3e, f).

**Fig. 4:**
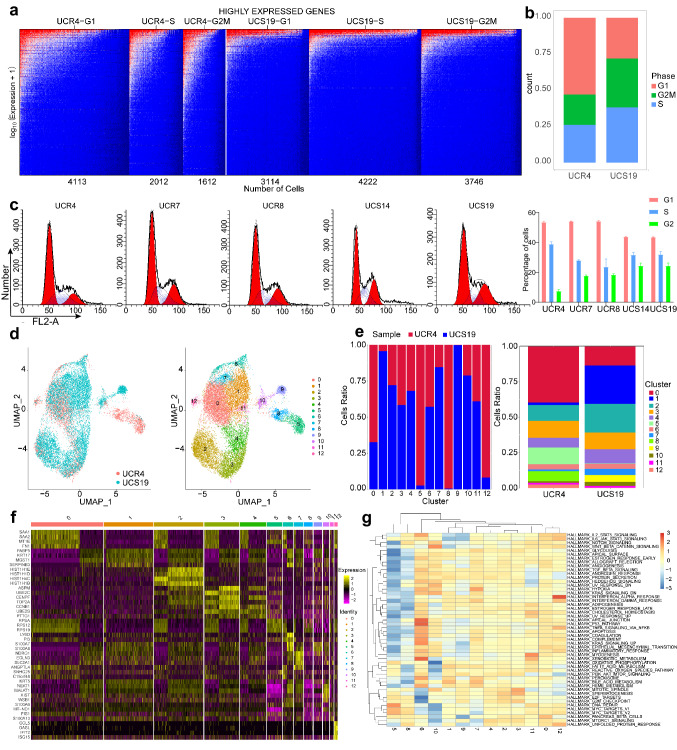
Radioresistant CR cell lines mainly accumulate in the G1 and S phases of the cell cycle. **a** Expression and cell cycle distribution of highly expressed genes in UCR4 and UCS19. **b** Cell cycle proportions in UCR4 and UCS19 according to the number of cells in Fig. 4a. **c**, Cell cycle distribution detected in primary cell lines using flow cytometry. **d** The UMAP plots demonstrating cell clusters and samples in UCR4 combined with UCS19. **e** Proportion of cells from UCR4 and UCS19 in each cell cluster, and proportion of cell clusters in UCR4 and UCS19 separately. **f** Heatmap showing expression levels of specific markers in cell clusters in UCR4 combined with UCS19. **g** Heatmap of hallmark pathways enriched in each cell cluster by GSVA

In general, cells in different cell cycle phases exhibit different radiosensitivity. Previous studies have shown that cells were most sensitive to irradiation during mitosis and in the G2 phase, less sensitive in the G1 phase and least sensitive in the latter S phase [22]. The CR cell lines were enriched in different cell cycle phases based on the expression levels of highly expressed genes (Fig. 4a). The proportion of cells in the G1 and G2/M phases in UCR4 was 53.16% and 20.83%, respectively, compared with 28.10% and 33.8% of cells in UCS19 (Fig. 4b). We verified the difference in cell cycle distribution of CR cell lines UCR4, UCR7, UCR8, UCS14, and UCS19, as well as traditional cell lines ME180 and C33A in vitro (Fig. 4c). Generally, radioresistant cells accumulated in the G1 and S phases, which concurred with previously reported results.

Next, the scRNA-seq results for UCR4 and UCS19 were combined to better understand the radiosensitivity of CC cells, yielding 13 cell clusters using the graph-based clustering method (Fig. 4d, e). Cell clusters 8, 5, 12, and 0 were associated with radioresistance, with the majority originating from UCR4, whereas cell clusters 9, 1, 7, and 10 were associated with radiosensitivity, with the majority originating from UCS19 (Fig. 4f, g). Highly expressed genes in cell cluster 8 were correlated with Myc signaling, while those in cluster 5 were enriched in the DNA repair pathway, which repaired DNA damage caused by irradiation. Highly expressed genes in cell cluster 12 were related to the IL6-JAK-STAT3 and IFN pathways, which were associated with immune response according to previous studies (Fig. 4g).

**Fig. 5:**
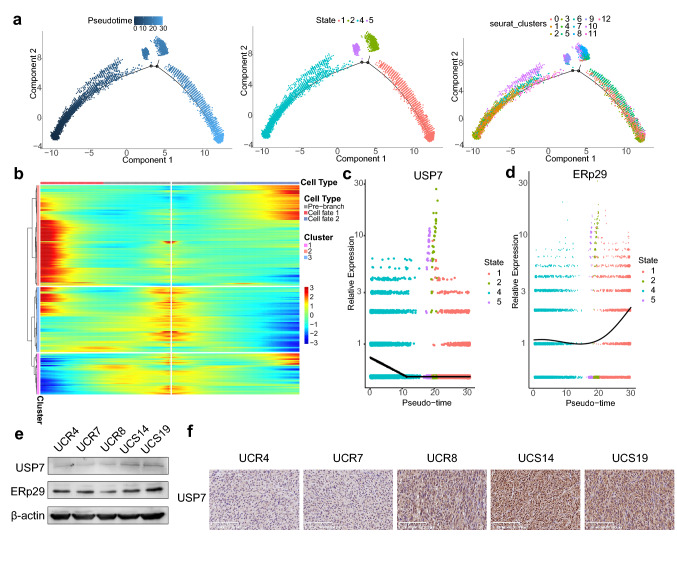
The transformation from radiosensitivity to radioresistance. **a** Cell cluster transition, cell state transition, and pseudo-trajectory of cell clusters. **b** Pseudotime heatmap displaying differentially expressed genes at the branch 1 according to BEAM analysis. Colors from blue to red indicate relative expression levels from low to high. **c** Expression of USP7 according to cell state. **d** Expression of ERp29 according to cell state. **e** Expression of USP7 and ERp29 in CR cell lines. **f** Expression of USP7 by IHC of xenografts. BEAM, branched expression analysis modeling

To clarify the transcriptional changes associated with radiosensitivity, unsupervised pseudo-time analysis was employed to reveal the gene expression trajectory. Most radioresistant cell clusters (5, 12, and 0) were addressed in state 1, whereas the majority of radiosensitive cell clusters were placed in state 4. Branched expression analysis modeling (BEAM) indicated that cell cluster 8 was at one end of the pseudotemporal trajectory, while cell cluster 9 was located at the other end (Fig. 5a). Kyoto Encyclopedia of Genes and Genomes (KEGG) enrichment analysis at branch 1 of the transformation to radioresistance revealed that genecluster 1 was enriched in oxidative phosphorylation and protein processing in the endoplasmic reticulum (ER), which were associated with oxidative stress (Fig. 5b, Online Resource 1. Figure 2a). Genecluster 3 was enriched in the cell cycle, FoxO signaling, and cellular senescence pathways (Fig. 5b, Online Resource 1. Figure 2b). Furthermore, cells at branch 1 showed increased expression of endoplasmic reticulum protein 29 (ERp29), a key component located in ER, which were crucial in transforming to a radioresistant phenotype, and decreased expression of human ubiquitin-specific protease 7 (USP7) in the forkhead boxO (FoxO) pathway (Fig. 5c, d) [23, 24]. ERp29 promoted the chemoresistance mediated by ER stress. Besides, oxidative stress induced the mono-ubiquitination and transcriptional activity of FoxO4 in radioresistant cells with decreased USP7, a deubiquitinating enzyme, thereby causing G0/S arrest and prevention of irradiation-induced cell death [23]. We verified that USP7 decreased in radioresistant cells while no significant difference in expression of ERp29 was observed (Fig. 5e, f, Online Resource 1. Figure 3).

## Discussion

Definitive chemoradiotherapy is the standard treatment for advanced CC, but the local relapse rate ranges from 17.3 to 33.1%, with most cases recurring within 2 years [8, 25, 26]. Previous studies reported that the median survival of patients with regional recurrence was 8 months with a 4-year survival rate of 10.7%, confirming that patients with local relapse had poor prognosis [27]. Human cancers are characterized by their histological complexity and genetical diversity. However, cancer cell lines undergo long-term clonal proliferation and adapt to culturing conditions, which prevents their generalization to PDTs [16]. The use of traditional cancer models (cell lines and animal models) is limited in both basic and clinical research, while precision medicine based on genomics benefits no more than 20% of patients with solid tumors [28]. Primary cell lines are a more appropriate option for studying cell biology and the unique characteristics of PDTs. However, cell senescence limits the life span and population doubling times of primary cell lines, resulting to low yields and restricting their applicability [20]. Compared with PDX and organoids, CR has advantages of utilizing various specimen sources and is relatively cheap and rapid, making it ideal for high-throughput screening in tumor research. CR cell lines that possess the same radiosensitivity as patients will be part of the next-generation living biobanks and human cancer model initiative programs launched by the American Association for Cancer Research [17].

In addition to the intrinsic limitations of cancer cell lines, most traditional cell lines used today are without detailed documentation for treatment. The cell lines are over-simplified for different therapies, which may lead to overtreatment, inefficiency, and even unnecessary side effects [29, 30]. The radioresistant and radiosensitive CR cell lines established in the current study maintained the characteristics of the original PDTs both in vitro and in vivo. Besides, their biomarkers expression and radiosensitivity were verified. Thus, the current study established a feasible approach to investigate radiosensitivity in CC, while maintaining intra-tumoral heterogeneity and characteristics consistent with the patients’ clinical information.

The results of scRNA-seq analysis revealed that a greater proportion of radioresistant CR cells aggregated in the G1 phase of cell cycle, which is resistant to radiation, while most radiosensitive cells were in the G2 phase, which is considered to be sensitive to radiation. Cyclins are proteins that activate specific CDKs, which are necessary for progression of the cell cycle [31].

A previous study reported that genes associated with the IFN-α response, IL2-STAT5, and IL6-JAK-STAT3 signaling pathways were enriched in tumors of patients with recurrent olfactory neuroblastoma [32]. The IL6-JAK-STAT3 signaling pathway promotes tumor invasive growth and suppresses the antitumor immune response. Moreover, IFN-α signaling after chronic activation induces tumor PD-L1 expression and dendritic cell exhaustion, thus providing a pro-tumorigenic microenvironment [33]. In the current study, the expression of genes associated with the IL6-JAK-STAT3 signaling pathways and IFN response changed significantly in the UCR4 cell line (Online Resource 1. Figure 1c). Therefore, targeting the IL6-JAK-STAT3 pathway and IFN-α response may inhibit tumor proliferation and activate the antitumor immune response to benefit patients with radioresistant CC.

Pseudo-time analysis of the combined UCR4 and UCS19 CR cell lines determined a branched gene expression trajectory related to transformation to a more radioresistant state. KEGG enrichment analysis revealed that oxidative stress, the cell cycle, and the FoxO signaling pathway played an important role in the reprogramming of radiosensitivity. The ER is the central organelle of protein folding, and trafficking, and ER stress contributes to many pathophysiological conditions caused by disturbances in the cell [34]. Radioresistant cells showed active oxidative-stress-related pathways with increased expression of genes associated with oxidative phosphorylation and ER stress, which mediated the survival of cells under stress caused by irradiation.

The subfamily of transcription factors of Fox gene family is widely expressed in cells and activated by a variety of growth factors and other stimulatory signals, including oxidative stress. The Fox genes regulates the specific expression of downstream molecules and cellular activity of the cell cycle, senescence, apoptosis, oxidative stress, stem cell differentiation, and tumor development and occurrence [35–37]. Increased cellular oxidative stress results in the mono-ubiquitination of FoxO4, which stimulates nuclear localization and raises transcriptional activity [23]. FoxO proteins promote tumor proliferation by inducing oxidative stress resistance and DNA damage repair and arrest cells in the G0/S phase [38, 39]. However, in response to oxidative stress, USP7 binds to FoxO4 to deubiquitinate it and inhibits the transcriptional activity. Radioresistant cells exhibited decreased expression of USP7, which prevented irradiation-induced cell death. Taken together, these findings suggest that USP7 may provide therapeutic targets for patients with recurrent CC.

## Conclusions

This study is the first to establish primary radioresistant and radiosensitive CC cell lines using CR and verify their characteristics and heterogeneity in vitro. Notably, the CR cell lines retained their tumorigenic phenotypes in vivo. With refinement of the CR technique and its combination with other advanced models (PDXs etc.), these cell lines may serve as a promising platform for cancer research, including studies exploring cancer biology, high-throughput drug screening, personalized treatment, and biobanking repositories. Radioresistant cells are in G1 and S phases and increase the FoxO signaling and oxidative stress pathway to support the cells after irradiation. This study infers that USP7 can be the potential radiosensitization targets after careful scientific researches in the future.

## Supplementary Information

Below is the link to the electronic supplementary material.

Supplementary file1 (PDF 1094 KB)

Supplementary file3 (XLSX 32 KB)

## Data Availability

The raw sequence data reported in this paper have been deposited in the Genome Sequence Archive in National Genomics Data Center, China National Center for Bioinformation/Beijing Institute of Genomics, Chinese Academy of Sciences (GSA-Human: HRA002566) that are publicly accessible at https://ngdc.cncb.ac.cn/gsa-human [40, 41].
