# Supplementary material for: Insights into the characteristics of primary radioresistant cervical cancer using single-cell transcriptomics
Source: Hum Cell. 2023 Mar 3;36(3):1135–46. doi: 10.1007/s13577-023-00882-x (PMC10110719; doi:10.1007/s13577-023-00882-x)
Supplement: Supplementary file 1 — Supplementary file1 (PDF 1094 KB) [file 13577_2023_882_MOESM1_ESM.pdf]

Title: Insights into the characteristics of primary radioresistant cervical cancer using single-cell transcriptomics

Journal: Human Cell

Author: Biyuan Xing[1,2a], Congli Pu[1,2a], Yunshang Chen[1,2a], Yuhan Sheng[1,2], Baofang Zhang[1,2], Jie Cui[3]*, Gang Wu[1,2]*, Yingchao Zhao[1,2]*

Author affiliations:

1 Cancer Center, Union Hospital, Tongji Medical College, Huazhong University of Science and Technology, Wuhan, 430022, China

2 Institute of Radiation Oncology, Union Hospital, Tongji Medical College, Huazhong University of Science and Technology, Wuhan, 430022, China

3 CAS Key Laboratory of Molecular Virology & Immunology, Institut Pasteur of Shanghai, Center for Biosafety Mega-Science, Chinese Academy of Sciences, Shanghai, China

a Biyuan Xing, Congli Pu and Yunshang Chen contribute equally to this work.

* Corresponding authors: Yingchao Zhao, Gang Wu and Jie Cui contribute equally to this work.

Yingchao Zhao MD. PhD.

2006xh0836@hust.edu.cn

Tel: +86 027-65655802

Fax: +86 027-65655802

Cancer Center, Union Hospital, Tongji Medical College, Huazhong University of Science and Technology, Wuhan, 430022, China and Institute of Radiation Oncology, Union Hospital, Tongji Medical College, Huazhong University of Science and Technology, Wuhan, 430022, China

Gang Wu MD. PhD.

xhzlwg@163.com

Cancer Center, Union Hospital, Tongji Medical College, Huazhong University of Science and Technology, Wuhan, 430022, China and Institute of Radiation Oncology, Union Hospital, Tongji Medical College, Huazhong University of Science and Technology, Wuhan, 430022, China

Jie Cui PhD.

jcui@ips.ac.cn

CAS Key Laboratory of Molecular Virology & Immunology, Institut Pasteur of Shanghai, Center for Biosafety Mega-Science, Chinese Academy of Sciences, Shanghai, China

Acknowledgements

This study was funded by the National Natural Science Foundation of China (Grant No. 81974463).

Materials and Methods

Raw disembarkation data quality statistics

The raw image data files obtained by high-throughput sequencing platform (Illumina HiSeqTM2500) were identified by CASAVA (Base Calling) analysis and converted into the original sequenced reads (Raw data), and the result was stored as FASTQ (referred to as fq) file format, which contained sequence information of Raw data and the corresponding sequencing quality information. Used FASTQ to log off Raw data quality for basic statistics.

Cell Ranger

Cell Ranger is an official software package from 10x genomics dedicated to the analysis of its single-cell transcriptome data. The FASTQ sequencing data was compared to the reference genome for UMI counting, and barcode filtering was performed according to the distribution of UMIs, and finally gene-cells were generated[1].

Cell Refiltration

After obtaining the gene expression matrix generated by Cell Ranger, we filtered the cells and genes again according to the number of gene detections, mitochondrial UMI ratio and other indicators to remove outliers, and use DoubleFinder to predict doublets in scRNA-seq data to remove the data. Concentrated multiple cells to ensure the reliability and accuracy of subsequent analysis results. Seurat (version 3.0.1)[2] was used for the procession quality control (QC). We detected 33694 genes and 9484 cells in UCR4 (Union Hospital Cervical Cancer Radioresistant 4). After filtering with nFeature_RNA, selecting greater than 200 and less than 6000, less than 30 of percent.mt, less than 5 of percent.HB, and genes expressing at least in 3 cells, we got 18550 genes and 7737 cells in UCR4 (Online Resource Fig.1a). As for UCS19 (Union Hospital Cervical Cancer Radiosensitive 19), we detected 33694 genes and 12010 cell. Through filtering with nFeature_RNA select greater than 200 and less than 5500, less than 30 of percent.mt, less than 5 of percent.HB, and genes expressing at least in 3 cells, we got 19544 genes and 11082 cells in UCS19 (Online Resource Fig.1e).

Dimensionality reduction and clustering

Subsequently, according to the dispersion of genes in samples, high-variable genes were selected for PCA analysis, and the appropriate number of PCs was selected through the gravel plot for subsequent dimensionality reduction and cluster. The class analysis and results were visualized by the t-distributed stochastic neighbor embedding (t-SNE) method and uniform manifold approximation and projection

(UMAP). UCR4 can be divided into 9 clusters, UCS19 can be divided into 8 clusters, and UCR4 combined with UCS19 can be divided into 13 clusters[3].

Differential gene and functional enrichment analysis

We identified the anchors using the FindIntegrationAnchors function, which took a list of Seurat objects as input, and used these anchors to integrate the UCR4 and UCS19 data with IntegrateData function. The input data for the genetic difference analysis was the raw UMI data obtained in the gene quantification. EdgeR package was used to analyze the significance of expressing differences, and adj_p $\leqslant$ 0.05 was the standard of differentially expressed genes (DEGs) between UCR4 and UCS19. As for the cluster marker genes, we used the FindAllmarker function in the Seurat package to identify the marker genes of each cluster, and analyzed the results of DEGs in each cluster compared with the other clusters. The screening standard was min.pct = 0.25 (pct.1 or pct.2 $\geqslant$ 0.25), p_val_adj < 0.05, avg_logFC > 0. Then, we used clusterProfiler package to perform Gene Ontology (GO) functional enrichment analysis on DEGs sets, and Kyoto Encyclopedia of Genes and Genomes (KEGG) pathway enrichment analysis to investigate the functional differences. DEGs in the clusters were investigated using GO and KEGG pathway analysis, and the FDR < 0.05 were considered significantly enriched[4].

Pseudo-time trajectory analysis

Based on the results of the Seurat analysis, the Monecle2 package (v.2.8.0) was further used for cell trajectory inference analysis. The trajectory changes of cells were fitted by cluster marker genes and visualized via 2D t-SNE graph. We also used the branched expression analysis modeling (BEAM) method to analyze the quasi-time-sorted cell data and the specified nodes, and then found DEGs related to branching[5].

Gene Set Variation Analysis (GSVA)

Getting ready of the expression matrix of UCR4 or UCS19 and gene set database, GSVA analysis was performed by GSVA function in order to find the DEGs sets in each cluster between UCR4 or UCS19 (Online Resource Fig.1c, g).

Single-cell CNV evaluation

We integrated normal cervical cancer data (GEO: GSE168652) into the combination of UCR4 and UCS19 using Harmony function and performed inferCNV analysis. We used the inferCNV package (version 1.4.0; https://github.com/broadinstitute/inferCNV/wiki) to discovery the CNVs in UCR4 and UCS19. The integrated normal cells were applied as the reference, with parameters including “denoise”,

default hidden markov model (HMM) settings, and a value of 0.1 for cut-off.

TF prediction in each cluster

We used the DoRothEA (http://bioconductor.org/ and https://github.com/saezlab/dorothea) to predict the TF in each cluster (Online Resource Fig.1d, h).

Statistical analysis

Statistical significance was determined by the Student's t-test or two-way ANOVA using GraphPad Prism 7.0 software (GraphPad Software, USA), and presented as *p<0.05, **p<0.01, ***p<0.001, ****p<0.0001. Results were represented as the mean ± standard deviation (SD) or mean ± standard error of the mean (SEM). Data shown were representatives of at least three independent experiments, which showed similar results.

Reference

1. Macosko EZ, Basu A, Satija R ,et al. Highly Parallel Genome-wide Expression Profiling of Individual Cells Using Nanoliter Droplets. Cell. 2015; 161:1202-1214.
2. Hao Y, Hao S, Andersen-Nissen E ,et al. Integrated analysis of multimodal single-cell data. Cell. 2021; 184:3573-3587.e3529.
3. Gu M, He T, Yuan Y, Duan S, Li X, Shen C. Single-Cell RNA Sequencing Reveals Multiple Pathways and the Tumor Microenvironment Could Lead to Chemotherapy Resistance in Cervical Cancer. Frontiers in Oncology. 2021; 11.
4. Chen EY, Tan CM, Kou Y ,et al. Enrichr: interactive and collaborative HTML5 gene list enrichment analysis tool. BMC Bioinformatics. 2013; 14:128.
5. Qiu X, Mao Q, Tang Y ,et al. Reversed graph embedding resolves complex single-cell trajectories. Nat Methods. 2017; 14:979-982.

Fig.1 The scRNA-seq reveals the features of radioresistant and radiosensitive CR cervical cancer cell lines

a, e, The t-SNE plot demonstrated main cluster types in UCR4 and UCS19, respectively. b, f, The expression of biomarkers in different clusters of UCR4 and UCS19, respectively. c, g, The GSVA analysis of every cluster in UCR4 and UCS19, respectively. d, h, The heatmap of transcriptional factors of UCR4 and UCS19, respectively.

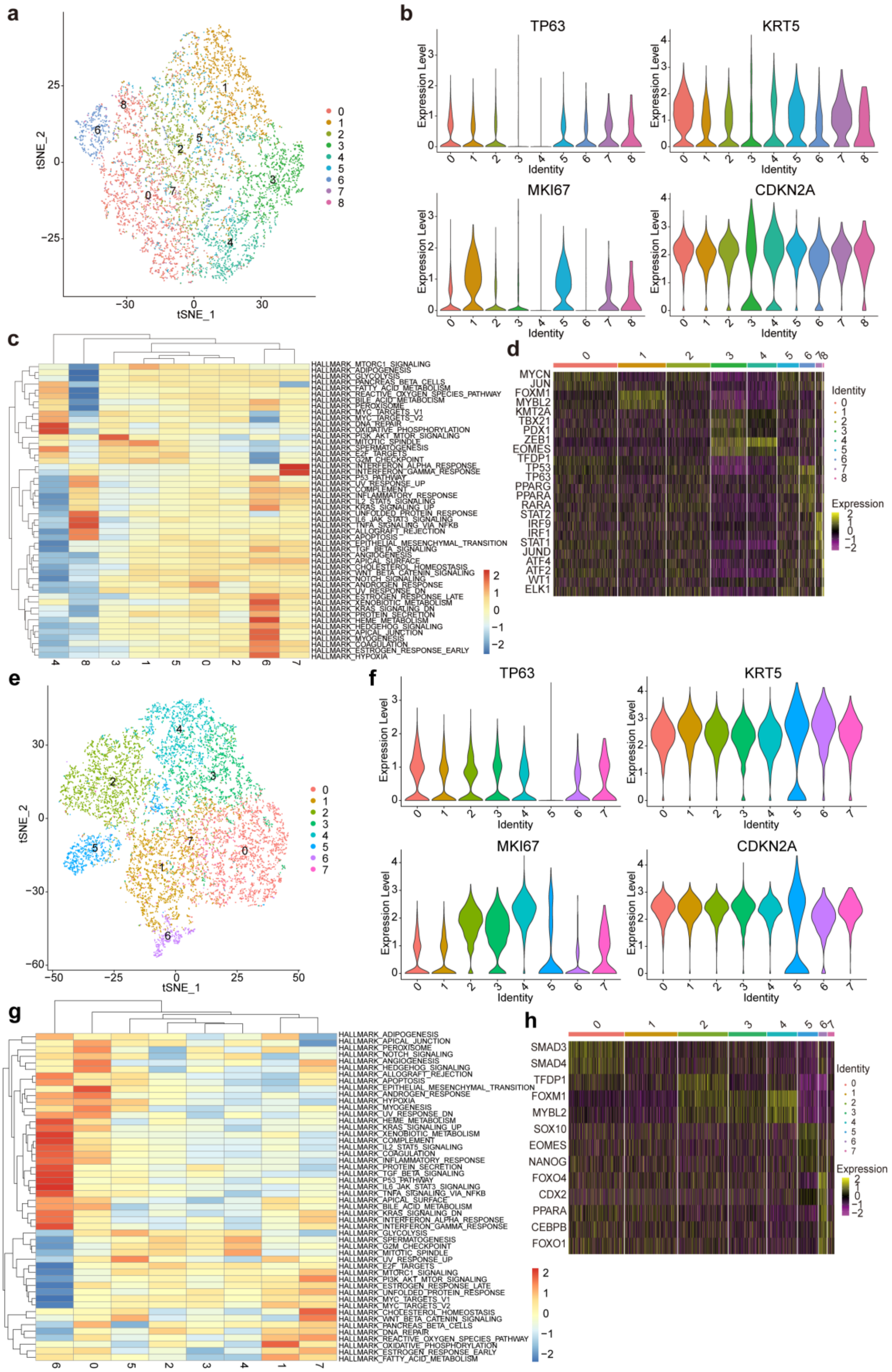
a
tSNE_1
tSNE_2
b
TP63
KRT5
MKI67
CDKN2A
Expression Level
Identity
c
HALLMARK_MTORC1_SIGNALING
HALLMARK_ADIPOGENESIS
HALLMARK_GLYCOLYSIS
HALLMARK_PANCREAS_BETA_CELLS
HALLMARK_FATTY_ACID_METABOLISM
HALLMARK_REACTIVE_OXYGEN_SPECIES_PATHWAY
HALLMARK_BILE_ACID_METABOLISM
HALLMARK_PEROXISOME
HALLMARK_MYC_TARGETS_V1
HALLMARK_MYC_TARGETS_V2
HALLMARK_DNA_REPAIR
HALLMARK_OXIDATIVE_PHOSPHORYLATION
HALLMARK_PI3K_AKT_MTOR_SIGNALING
HALLMARK_MITOTIC_SPINDLE
HALLMARK_SPERMATOGENESIS
HALLMARK_E2F_TARGETS
HALLMARK_G2M_CHECKPOINT
HALLMARK_INTERFERON_ALPHA_RESPONSE
HALLMARK_INTERFERON_GAMMA_RESPONSE
HALLMARK_P53_PATHWAY
HALLMARK_UV_RESPONSE_UP
HALLMARK_COMPLEMENT
HALLMARK_INFLAMMATORY_RESPONSE
HALLMARK_IL2_STAT5_SIGNALING
HALLMARK_KRAS_SIGNALING_UP
HALLMARK_UNFOLDED_PROTEIN_RESPONSE
HALLMARK_IL6_JAK_STAT3_SIGNALING
HALLMARK_TNFA_SIGNALING_VIA_NFKB
HALLMARK_ALLOGRAFT_REJECTION
HALLMARK_APOPTOSIS
HALLMARK_EPITHELIAL_MESENCHYMAL_TRANSITION
HALLMARK_TGF_BETA_SIGNALING
HALLMARK_ANGIOGENESIS
HALLMARK_APICAL_SURFACE
HALLMARK_CHOLESTEROL_HOMEOSTASIS
HALLMARK_WNT_BETA_CATENIN_SIGNALING
HALLMARK_NOTCH_SIGNALING
HALLMARK_ANDROGEN_RESPONSE
HALLMARK_UV_RESPONSE_DN
HALLMARK_ESTROGEN_RESPONSE_LATE
HALLMARK_XENOBIOTIC_METABOLISM
HALLMARK_KRAS_SIGNALING_DN
HALLMARK_PROTEIN_SECRETION
HALLMARK_HEME_METABOLISM
HALLMARK_HEDGEHOG_SIGNALING
HALLMARK_APICAL_JUNCTION
HALLMARK_MYOGENESIS
HALLMARK_COAGULATION
HALLMARK_ESTROGEN_RESPONSE_EARLY
HALLMARK_HYPOXIA
d
MYCN
JUN
FOXM1
MYBL2
KMT2A
TBX21
PDX1
ZEB1
EOMES
TFDP1
TP53
TP63
PPARG
PPARA
RARA
STAT2
IRF9
IRF1
STAT1
JUND
ATF4
ATF2
WT1
ELK1
Identity
Expression
e
tSNE_1
tSNE_2
f
TP63
KRT5
MKI67
CDKN2A
Expression Level
Identity
g
HALLMARK_ADIPOGENESIS
HALLMARK_APICAL_JUNCTION
HALLMARK_PEROXISOME
HALLMARK_NOTCH_SIGNALING
HALLMARK_ANGIOGENESIS
HALLMARK_HEDGEHOG_SIGNALING
HALLMARK_ALLOGRAFT_REJECTION
HALLMARK_APOPTOSIS
HALLMARK_EPITHELIAL_MESENCHYMAL_TRANSITION
HALLMARK_ANDROGEN_RESPONSE
HALLMARK_HYPOXIA
HALLMARK_MYOGENESIS
HALLMARK_UV_RESPONSE_DN
HALLMARK_HEME_METABOLISM
HALLMARK_KRAS_SIGNALING_UP
HALLMARK_XENOBIOTIC_METABOLISM
HALLMARK_COMPLEMENT
HALLMARK_IL2_STAT5_SIGNALING
HALLMARK_COAGULATION
HALLMARK_INFLAMMATORY_RESPONSE
HALLMARK_PROTEIN_SECRETION
HALLMARK_TGF_BETA_SIGNALING
HALLMARK_P53_PATHWAY
HALLMARK_IL6_JAK_STAT3_SIGNALING
HALLMARK_TNFA_SIGNALING_VIA_NFKB
HALLMARK_APICAL_SURFACE
HALLMARK_BILE_ACID_METABOLISM
HALLMARK_KRAS_SIGNALING_DN
HALLMARK_INTERFERON_ALPHA_RESPONSE
HALLMARK_INTERFERON_GAMMA_RESPONSE
HALLMARK_GLYCOLYSIS
HALLMARK_SPERMATOGENESIS
HALLMARK_G2M_CHECKPOINT
HALLMARK_MITOTIC_SPINDLE
HALLMARK_UV_RESPONSE_UP
HALLMARK_E2F_TARGETS
HALLMARK_MTORC1_SIGNALING
HALLMARK_PI3K_AKT_MTOR_SIGNALING
HALLMARK_ESTROGEN_RESPONSE_LATE
HALLMARK_UNFOLDED_PROTEIN_RESPONSE
HALLMARK_MYC_TARGETS_V1
HALLMARK_MYC_TARGETS_V2
HALLMARK_CHOLESTEROL_HOMEOSTASIS
HALLMARK_WNT_BETA_CATENIN_SIGNALING
HALLMARK_PANCREAS_BETA_CELLS
HALLMARK_DNA_REPAIR
HALLMARK_REACTIVE_OXYGEN_SPECIES_PATHWAY
HALLMARK_OXIDATIVE_PHOSPHORYLATION
HALLMARK_ESTROGEN_RESPONSE_EARLY
HALLMARK_FATTY_ACID_METABOLISM
h
SMAD3
SMAD4
TFDP1
FOXM1
MYBL2
SOX10
EOMES
NANOG
FOXO4
CDX2
PPARA
CEBPB
FOXO1
Identity
Expression

Fig.2 KEGG enrichment analysis of genes at branch 1 of turning to radioresistant cells

a, KEGG analysis of genes in genecluster 1. b, KEGG analysis of genes in genecluster 3.

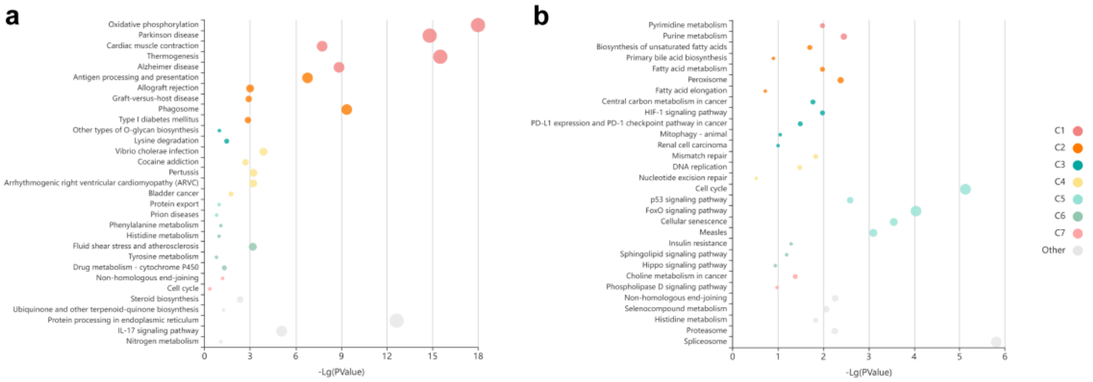

Fig.3 Expression of ERp29 by IHC of xenografts.

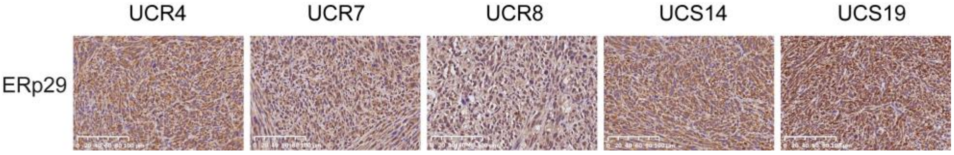

Table 1. STR loci of CR cell lines and peripheral blood of patients.

Loci	UCR4		UCR7		UCR8		UCS14		UCS19	
	Patient	CRC	Patient	CRC	Patient	CRC	Patient	CRC	Patient	CRC
D5S818	10, 12	10, 12	11	11	7, 12	7, 12	10, 12	10, 12	9, 13	9, 13
D13S317	8	8	8, 13	8, 13	11	11	11, 13	11, 13	8, 9	8, 9
D7S820	10, 12	10, 12	8, 12	8, 12	8, 11	8	9	9	9.1, 10	9.1, 10
D16S539	9, 12	9, 12	9	9	12	12	9	9	9, 12	9, 12
VWA	16, 18	16, 18	16, 17	16, 17	16, 17	16	17, 19	17, 19	14, 16	14, 16
TH01	9	9	6, 7	6	9	9	7, 9	7, 9	9	9
AMEL	X	X	X	X	X	X	X	X	X	X
TPOX	8, 11	8, 11	8, 11	8, 11	8, 11	8, 8	11	11	8	8
CSF1PO	11, 12	11, 12	10, 11	10, 11	11, 12	11, 12	11	11	12	12
D12S391	15, 18	15, 18	22	22	20	20	20, 26	20, 26	20, 23	20, 23
FGA	22, 26	22, 26	19, 24	19, 24	24, 26	24, 26	18, 23	23	20, 26	20, 26
D2S1338	23, 24	23, 24	17, 19	17, 19	19, 23	19	23	23	21, 24	21, 24
D21S11	29, 31.2	29, 31.2	30, 31.2	30, 31.2	31	31	30.2, 31.2	31.2	31	31
D18S51	13, 14	13, 14	16	16	13, 18	13, 18	16, 17	16, 17	15, 16	15, 16
D8S1179	12, 13	12, 13	10, 13	10, 13	10	10	13, 14	13, 14	12, 14	12, 14
D3S1358	15, 17	15, 17	15, 17	15, 15	15	15	15	15	16	16
D6S1043	11, 14	11, 14	13, 14	13, 14	14, 18	14	12	12	10, 13	10, 13
PENTAE	14	14	20, 21	20, 21	15, 17	15	10, 12	10, 12	15, 16	15, 16
D19S433	14, 15	14, 15	13	13	13, 14	13	13.2, 14.2	14.2	13.2, 14	13.2, 14
PENTAD	9, 12	9, 12	10, 11	10, 11	10, 13	10, 13	8, 10	8, 10	9, 16	9, 16
D1S1656	15, 17.3	15, 17.3	11, 16	11, 16	16, 17	16, 17	11, 13	11, 13	16, 17	16, 17

CRC, conditional reprogramming cell
